# Navigating Hyperhemolysis in Sickle Cell Disease: Insights from Literature

**DOI:** 10.3390/diagnostics15141835

**Published:** 2025-07-21

**Authors:** Sruthi Vellanki, Nishanth Thalambedu, Anup Kumar Trikannad Ashwini Kumar, Sravya Vellanki, Medhavi Honhar, Rachel Hendrix, Denese Harris, Mamatha Gaddam, Sunny R. K. Singh, Shivi Jain, Muthu Kumaran, Cesar Gentille, Ankur Varma

**Affiliations:** 1 Division of Hematology-Oncology, University of Arkansas for Medical Sciences, Little Rock, AR 72205, USA; nthalambedu@uams.edu (N.T.); rjhendrix@uams.edu (R.H.); harrisdenesed@uams.edu (D.H.); mgaddam@uams.edu (M.G.); srsingh@uams.edu (S.R.K.S.); mvkumaran@uams.edu (M.K.); cgentille@uams.edu (C.G.); 2 Division of Myeloma, University of Arkansas for Medical Sciences, Little Rock, AR 72205, USA; atrikannad@uams.edu; 3 Division of Internal Medicine, Willis Knighton Health System, Shreveport, LA 71103, USA; svellanki@wkhs.com; 4 Division of Pediatric Hematology Oncology, Arkansas Children’s Hospital, Little Rock, AR 72205, USA; mhonhar@uams.edu; 5 Division of Hematology, Oncology, and Cell Therapy, Rush University Medical Center, Chicago, IL 60612, USA; shivi_jain@rush.edu

**Keywords:** sickle cell disease, anemia, hyperhemolysis, eculizumab

## Abstract

Sickle cell disease (SCD) is a prevalent genetic disorder caused by a mutation in the beta-globin gene. Hyperhemolysis (HS) is a severe complication involving the rapid destruction of both transfused and endogenous red blood cells, commonly found in SCD. This literature review explores the clinical presentation, diagnosis, pathogenesis, and management of HS in SCD. HS can manifest acutely or in a delayed manner, complicating diagnosis due to overlapping symptoms and varying reticulocyte responses. Immunohematological assessments often reveal delayed positivity in direct antiglobulin tests and antibody screens. HS typically presents severe anemia, jaundice, hemoglobinuria, and hemodynamic instability. Diagnostic markers include elevated bilirubin and lactate dehydrogenase levels alongside a reduced reticulocyte count. The management of HS is primarily empirical, with no clinical trials to support standardized treatment protocols. First-line treatments involve steroids and intravenous immunoglobulins (IVIG), which modulate immune responses and mitigate hemolysis. Refractory cases may require additional agents such as rituximab, eculizumab, tocilizumab, and, in some instances, plasma exchange or erythropoietin-stimulating agents. Novel therapeutic approaches, including bortezomib and Hemopure, have shown promise but require further investigation. Current management strategies are empirical, underscoring the need for robust clinical trials to establish effective treatment protocols that ultimately improve outcomes for SCD patients experiencing HS.

## 1. Introduction

Hyperhemolysis (HS) is a severe and often life-threatening condition characterized by the accelerated destruction of red blood cells, leading to anemia and other associated complications. Initially identified in patients with sickle cell disease (SCD), it can also occur in other hemolytic conditions. HS may manifest during a hemolytic crisis or as a delayed reaction following blood transfusion, complicating the clinical management of patients with chronic hemolytic anemias [1,2,3].

Initial reports focused on its occurrence in SCD patients undergoing blood transfusions. Instead of the expected increase in hemoglobin levels, patients experienced a paradoxical drop due to the destruction of both transfused and native red blood cells. Over the past few decades, significant research has aimed to unravel the mechanisms underlying HS and develop effective management strategies.

HS is of significant clinical importance due to its severe impact on patients’ health. It leads to profound anemia, which can be life-threatening if not managed promptly and effectively. This condition also complicates transfusion therapy, which is a cornerstone of treatment for many patients with hemolytic disorders. Understanding HS is crucial for improving patient outcomes, especially in those with SCD, thalassemia, and other hemolytic anemias.

A comprehensive literature search was conducted using major scientific databases, including PubMed and Embase, with keywords such as “sickle cell”, “anemia”, “hemolytic anemia”, and “hyperhemolysis”. The searches were filtered for English language publications and sorted by the most recent publications to ensure the inclusion of the latest research findings. The identified studies were reviewed and analyzed systematically. The inclusion criteria were based on the relevance of the study to the topic of HS, the quality of the research, and the contribution to the understanding of HS—the review process aimed to ensure a thorough and unbiased synthesis of the available evidence.

This literature review focuses on several key aspects of HS: exploring the underlying mechanisms that lead to HS, including immune and non-immune factors; describing the signs and symptoms of HS; and reviewing the current approaches to managing HS. Our review aims to provide a detailed and comprehensive overview of HS, highlighting its significance in clinical practice and the ongoing challenges in its management. By identifying the key areas of research and current gaps in knowledge, this review seeks to contribute to the advancement of understanding and the treatment of HS, ultimately improving patient care and outcomes.

## 2. Pathogenesis

**Figure 1:**
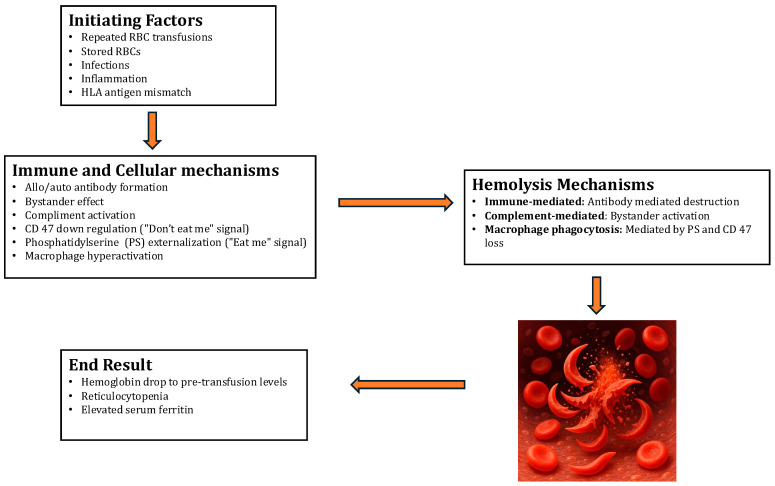
Pathogenesis of HS in Sickle Cell Patients.

The pathogenesis of Hyperhemolytic Syndrome (HS) involves several intricate mechanisms, but a comprehensive understanding remains elusive. Multiple theories (Table 1, Figure 1) have been proposed to elucidate the mechanisms behind HS. One notable feature is the destruction of both the patient’s own (autologous) and donated red blood cells (RBCs). Sickle cell patients are particularly vulnerable to multiple transfusions, which can lead to the development of alloantibodies against the RBC antigens in the donor blood units. Although the levels of these alloantibodies diminish over time, subsequent exposure to the same RBC antigens triggers an anamnestic reaction, resulting in the destruction of both autologous and donor RBCs. While marrow RBC precursors may also be affected, this occurrence is relatively rare among sickle cell patients. Moreover, HS tends to recur in the same patient following subsequent blood transfusions, suggesting an underlying genetic predisposition akin to a secondary immune response.

Hyperactive macrophages are thought to cause RBC destruction primarily. Various factors can contribute to RBC injury, including osmotic shock, oxidative stress, or ATP depletion. These factors stimulate the production of prostaglandin E2, leading to the activation of calcium-permeable channels and the subsequent influx of calcium ions into the RBC cytoplasm. This cascade of events also involves the opening of calcium-sensitive potassium channels, causing intracellular potassium loss. Additionally, enzymes such as scramblase, activated by calcium and ceramide derived from stressors like osmotic shock, facilitate the movement of phosphatidylserine (PS) to the outer leaflet of the RBC membrane. In normal conditions, phospholipids are asymmetrically distributed across the RBC membrane, with PS predominantly located in the inner leaflet. However, in sickle cell anemia (SCA), oxidative stress leads to a distortion known as “flip-flop”, with PS predominantly exposed on the outer leaflet. Exposure to PS acts as a signal for macrophage phagocytosis, akin to the process of apoptosis in nucleated cells [1,4].

The concept of bystander hemolysis, as introduced by Garratty in 2008, addresses an indirect mechanism of red cell destruction that is particularly relevant in the context of sickle cell disease (SCD). This theory hinges on the increased fragility and abnormal morphology of sickle-shaped red cells, which are inherently more prone to hemolysis. In patients with SCD, repetitive blood transfusions are common and can lead to an immunological phenomenon where antibodies are produced not only against transfused red cells but also against platelets and human leukocyte antigens (HLA). These antibodies may also target foreign protein epitopes that are not directly involved in the initial immune response [2].

Bystander hemolysis occurs when these antibodies, while targeting one antigen, inadvertently lead to the destruction of neighboring red cells that do not necessarily express the target antigen. This results in a significant increase in hemolysis, not directly caused by the immune system attacking the red cells for their antigens but, rather, as collateral damage during other immune responses. This mechanism significantly complicates the management of transfusion in SCD patients, as it can lead to an unexpected increase in hemolysis rates following transfusions, complicating patient outcomes and treatment protocols [3,4,5]. Furthermore, bystander complement activation on the surfaces of autologous RBCs, triggered by antibodies against donor antigens in the patient’s serum, may contribute to hemolysis. A defective regulation of the membrane attack complex (MAC) on the RBCs of SCA patients could also lead to the complement-mediated destruction of both transfused and autologous RBCs. This dysregulation affects all RBC precursors expressing the target antigens, resulting in reticulocytopenia and a scarcity of red cell precursors in some cases [4].

Win et al. provided insights into another significant contributor to HS in SCD—macrophage activation. Their studies suggest that red cells from SCD patients exhibit increased adhesion to macrophages compared to normal hemoglobin A (HbA) red cells. This increased adhesion is problematic because it facilitates phagocytosis and the subsequent destruction of sickle cells and reticulocytes by macrophages. This theory is supported by elevated serum ferritin (SF) levels, an indirect indicator of increased macrophage activity. Consequently, there is a significant drop in hemoglobin levels below those before transfusion and a decrease in reticulocyte count [6,7]. The role of macrophage activation in acute post-transfusion hyperhemolysis syndrome (PTHS) includes direct erythrophagocytosis. Contributing factors to macrophage activation may include pathways triggering activation, an increased expression of adhesion molecules on RBCs, and an inflammatory milieu. Notably, the interaction between CD47 on RBCs and SIRP-α on macrophages typically sends a “don’t eat me" signal, preventing phagocytosis. However, alterations in CD47 expression or conformation might shift this balance, prompting macrophages to destroy RBCs. This effect could be exacerbated in stored RBCs and sickle cells, which exhibit reduced CD47 expression [5,8]. Furthermore, sickle RBCs and reticulocytes exhibit an increased expression of adhesion molecules such as phosphatidylserine and α4β1, enhancing their binding to vascular cell adhesion molecules and promoting interactions with macrophages. Additionally, the macrophage-exclusive scavenger protein CD163, which binds to hemoglobin–haptoglobin complexes, may further promote macrophage activation during RBC destruction [7,8] [Table 2].

In cases of HS with detectable alloantibodies, it is believed that an “innocent bystander” effect occurs, where antibody–antigen complexes attach to the patient’s own red cells, sometimes involving autoantibodies. In the alloantibody-negative form, the exact mechanisms of severe hemolysis are less understood, though theories include direct interactions between red cells and activated macrophages, as well as the involvement of cytokine and complement pathways, underscoring the immune system’s significant role in this condition [8]. Among these are antibody-mediated immune hemolysis and bystander hemolysis mechanisms. In scenarios where no antibodies are detectable in acute PTHS, macrophage activation is a likely alternative mechanism. This hypothesis is reinforced by increased serum ferritin, a marker of macrophage activity, during episodes of PTHS [9]. In particular, the bystander effect hypothesizes that complement activation, to which sickled erythrocytes are particularly vulnerable, might contribute to autologous RBC destruction in delayed, antibody-mediated PTHS, although direct evidence is lacking.

Some red blood cells express HLA, and the presence of HLA antibodies can lead to both acute and delayed hemolytic transfusion reactions (DHTR). The formation of HLA antibodies is notably common among patients with sickle cell disease. Previous studies proposed that transfused red cells expressing HLA are targeted and destroyed by HLA-mediated immune mechanisms involving hyperactive macrophages, while those transfused cells that do not express HLA are destroyed via a bystander mechanism. Additionally, fever is frequently observed in cases of HS, potentially indicating an infection that could activate macrophages, further contributing to HS [10].

## 3. Clinical Presentation of HS

HS is a specific type of delayed hemolytic transfusion reaction (DHTR) primarily observed in SCD. It can appear in both acute and delayed forms. Acute cases appear within the first week after a transfusion, while delayed cases occur afterward. In these delayed cases, a positive direct antiglobulin test (DAT) and the detection of RBC alloantibodies are common, complicating the diagnosis. There is an overlap of symptoms between the acute and delayed forms, highlighting the importance of meticulous evaluation and a tailored approach to both the diagnosis and management of transfusion reactions in these patients [11,12,13].

During a DHTR, reticulocyte counts may vary; some patients may experience an appropriate increase in reticulocytes, whereas others may show a decrease. Older patients often have a lower reticulocyte response, likely due to diminished bone marrow capacity from repeated infarctions and decreased red blood cell production, potentially prolonging the DHTR.

The immunohematological assessment of SCD during a DHTR can be challenging; initial antibody screens and DATs may be negative despite significant hemolysis. It may take about four days from the lowest hemoglobin reading to the first positive DAT or antibody screen, sometimes due to delayed testing by healthcare providers. While DAT positivity is associated with hemolysis, it does not consistently correlate with the severity of symptoms and must be interpreted carefully. A strongly positive DAT typically suggests the possibility of detecting alloantibodies later. It is important to note, however, that a positive DAT is not necessary to diagnose a DHTR, and the presence of alloimmunization does not guarantee a DHTR will occur [11,14].

Distinguishing HS from other types of delayed transfusion reactions requires careful consideration. The hallmark of HS includes a decrease in hemoglobin levels below the levels before transfusion, accompanied by a low reticulocyte count and no detectable RBC alloantibodies. This differs from a typical DHTR, which usually shows an increase in reticulocytes and stable hemoglobin levels with detectable alloantibodies.

The clinical manifestations of HS may include significant worsening of anemia, jaundice, and hemoglobinuria, along with signs of hemodynamic instability such as tachycardia, hypotension, and fatigue. Early signs can include fever and pain, typical of sickle cell crises.

Diagnostic indicators of HS include an increase in bilirubin, elevated lactate dehydrogenase (LDH) levels, and hemoglobinuria. Moreover, there is a reduction in the absolute reticulocyte count compared to the patient’s typical levels, suggesting a suppression or destruction of the bone marrow response [15]. Hemoglobin fraction tests often show a drop in HbA% (as donor cells are destroyed) accompanied by an increase in HbS%.

## 4. Management

Management of HS presents a complex clinical challenge marked by diverse pathophysiological mechanisms and variable treatment responses. Typically, it manifests as a severe hemolytic crisis characterized by an unexpected worsening of anemia following a blood transfusion. Despite advancements in understanding the immunological and hematological processes driving HS, its optimal management remains empirical, lacking evidence-based guidelines to guide clinical decisions. Therefore, effective strategies require a holistic approach that integrates early recognition, the prudent use of immunosuppressive agents, and the exploration of novel therapeutic modalities tailored to individual patient needs. To deepen comprehension, we conducted a comprehensive literature review to uncover previous publications, offering insights into the historical context and contemporary understanding of HS management.

The majority of HS cases occurred in late adolescence to middle-aged sickle cell patients with no clear gender discrimination. The trigger for HS in most cases was either a simple blood transfusion or exchange transfusion, and the time to develop HS after the trigger was observed to be 2–7 days. The timing of HS development reported in the prior studies was consistent with the presumed pathophysiology of macrophage-mediated acute HS and antibody-mediated delayed HS. Although there is a considerable overlap in the timing and lab results, posing difficulties in delineating the acute vs. delayed types, the crucial point for optimal outcomes remains to be the early identification of hyperhemolysis and its appropriate management.

Management relies on the early identification of HS. Once HS, either acute or delayed, is diagnosed via the laboratory findings, attempts should be made to limit blood transfusions. The current management of HS is primarily empirical, with no clinical trials examining the efficacy of various immunosuppressive regimens. Standard first-line treatments used in almost all patients included steroids and intravenous immunoglobulins (IVIG). These agents mitigate hemolysis by expediting the clearance of endogenous pathogenic autoantibodies, modulating the immune response, and inhibiting components of the complement cascade. Treatment should be carefully monitored as they impose specific challenges for SCD patients, such as rebound pain upon steroid cessation and the hyperviscosity risk secondary to IVIG. The benefits of starting steroids should always be weighed with the risks associated with it, especially with stopping abruptly. A prolonged tapering schedule of steroids is usually recommended.

Rituximab, a CD-20 monoclonal antibody, has been utilized in cases refractory to first-line agents and can induce varying levels of immunosuppression, which may endure beyond the initial treatment course. Various dosing schedules have been used in the literature, including low-dose (100 mg IV weekly for four weeks) and standard-dose (375 mg/m^2^ weekly × 4). This is a matter of potential concern in SCD, particularly when there is concurrent functional asplenia due to increasing the risk of infections [16].

Third-line agents include a variety of options. Eculizumab, a human monoclonal antibody, has been used in multiple patients, as mentioned in Table 3. It targets complement protein C5 and has demonstrated its effectiveness in treating HS by functioning as a C5-convertase inhibitor. This action prevents the division of C5 into C5b, thereby blocking the formation of the terminal complex C5b-9 in the complement cascade. Complement component C3 plays a central role in the complement cascade and in hemolytic anemias such as HS. While anti-C5 therapy (e.g., eculizumab) is often highlighted due to its clinical use, it is the deposition of C3b on RBC membranes that marks them for extravascular destruction via macrophage phagocytosis in the spleen and liver. In fact, C3 fragment deposition often precedes and facilitates C5 activation. Emphasis should be placed on the significant risk of meningococcal infections, highlighting the necessity of vaccination to mitigate the likelihood of contracting meningococcal disease. The therapeutic effectiveness of eculizumab in atypical hemolytic uremic syndrome (aHUS) and paroxysmal nocturnal hemoglobinuria (PNH) was measured using special assays that track eculizumab trough levels and CH 50 activity with established thresholds in these conditions. Eculizumab serum trough concentrations of >99 µg/mL in aHUS and >35 µg/mL in PNH as well as CH50 being <10% of the lower limit of normal suggests an adequate suppression of the terminal complement pathway. However, these assays were not tested in HS and conclusions were extrapolated from other disease subtypes for better clinical monitoring in HS [17,18,19,20]. Soluble C5b-9 (sC5b-9), a novel biomarker to monitor the activity of terminal complement pathway activation, can be used in diagnosis and disease monitoring because Eculizumab is known to block the conversion of C5 to its active components C5a and C5b, thereby decreasing the levels of sC5b-9 when elevated. There is no clear guidance on the eculizumab dosing schedule, but the consensus is to use 900 mg/week until an improvement in the hemolysis is noted.

Tocilizumab, an activity blocker for Interleukin-6 (IL-6), a critical inflammatory/immune-mediated cytokine in macrophage activation, was used as a salvage treatment in a few cases of hyperhemolysis syndrome in sickle cell disease [3]. The dose used was 8 mg/kg/day for a duration varied from one to four days. The clinical improvement of hemolysis was noted in all these studies, suggesting the need to explore its role in this context. Plasma exchange was tried as a supportive measure in two cases of HS in sickle cell disease patients, refractory to standard options. It predominantly works by removing the auto and alloantibodies there by temporarily attenuating the process and allowing other treatments to take effect, thereby ultimately leading to the resolution of life-threatening hemolysis [27].

The role of erythropoietin-stimulating agents (ESA) has been tested in a few settings. It can have benefits in the realm of HS even with normal/elevated erythropoietin levels, which were considered inappropriately low for the degree of anemia. For the same reason, IV iron can be supplemented with ESA to maximize innate erythropoiesis, but caution should be advised in SCD patients who are already iron-overloaded due to multiple prior transfusions [8,23,24].

Epstein et al. tried two novel therapeutic approaches to treat HS in a SCD patient after the first-line treatments (Steroids, Rituxan, and ESA) failed to show improvement. Bortezomib, a proteasome inhibitor, was initiated at 1.3 mg/m^2^/dose every 72 h up to four doses. The patient received one unit of Hemopure for declining Hb levels four days after starting bortezomib, which improved the Hb. Following this, the patient received further doses of bortezomib, which halted further hemolysis and improved Hb [8]. The rationale for using bortezomib was to decrease their immune response to avoid fatality from hemolysis. It was thought to reduce the inflammatory and infiltrative functions of macrophages by inhibiting cytokines and cellular adhesion molecules mirroring our presumed macrophage-mediated pathogenesis in acute HS. Additionally, Hemopure, a polymerized bovine hemoglobin substitute that is universally compatible and does not require donor–recipient compatibility testing before administration, was used to prevent tissue hypoxia from dangerously low Hb levels. It is important to note that the Hemopure product interferes with colorimetric assays for liver function tests, making these tests difficult to interpret.

**Figure 2:**
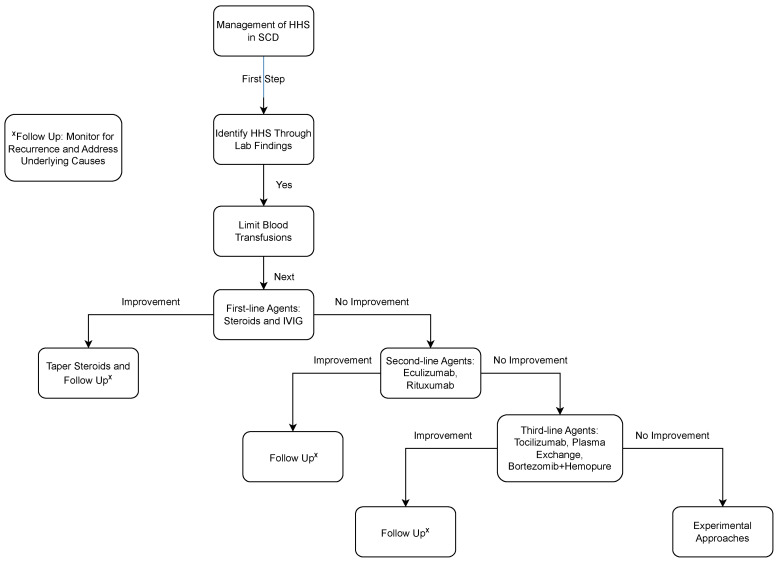
Management of Hyperhemolysis Syndrome (HS) in sickle cell disease algorithm.

Overall, managing HS in SCD presents significant challenges, underscored by its empirical nature and the absence of robust clinical trials examining specific treatment regimens. Current strategies primarily rely on the early identification of HS and the judicious use of immunosuppressive agents such as steroids, IVIG, and rituximab. While these treatments offer potential benefits in mitigating hemolysis, they also pose unique risks and complexities, necessitating vigilant monitoring and careful consideration of individual patient factors. Novel approaches, including Eculizumab, tocilizumab, plasma exchange, and erythropoietin stimulating agents, have shown promise in select cases but require further investigation to delineate their efficacy and safety profiles. A suggested pathway for the management of HS is shown in Figure 2. Moving forward, collaborative efforts between clinicians and researchers are essential to advance our understanding of HS pathophysiology and optimize therapeutic approaches, ultimately improving outcomes for patients with SCD experiencing HS.

## 5. Current Guidelines

The American Society of Hematology (ASH) defined a DHTR as a significant drop in hemoglobin within 21 days after a transfusion, along with peripheral blood hemolysis indicators and HS involving a rapid decline in hemoglobin levels post-transfusion. The guideline panel suggests using immunosuppressive therapy (IVIG, steroids, rituximab, and/or eculizumab) instead of no therapy in individuals with sickle cell disease (all genotypes) who experience a delayed hemolytic transfusion reaction (DHTR) and ongoing hyperhemolysis.

IVIG and high-dose steroids are recommended first-line agents, with eculizumab considered as a second-line option. Rituximab is primarily used to prevent additional alloantibody formation in patients who may require further transfusion.

Steroid therapy should be tapered cautiously to avoid vaso-occlusive episodes. Further transfusions should generally be avoided unless patients are experiencing life-threatening anemia with ongoing hemolysis, in which case extended matched red cells should be considered. It also emphasized the importance of supportive care, including erythropoietin with or without IV iron and a shared decision-making process involving patients throughout their treatment journey [28].
**ASH Guidelines for DHTR and HS Management in SCD**Definitions of DHTR and HSDHTR: Significant drop in hemoglobin within 21 days post-transfusion, along with peripheral blood hemolysis indicators.
HS: Rapid decline in hemoglobin levels post-transfusionFirst-line AgentsIVIG and high-dose steroids. Caution should be exercised while steroid tapering to prevent vaso-occlusive episodesSecond-line AgentsEculizumabRituximabPrimarily to prevent additional alloantibody formationTransfusion StrategyTransfuse only in life-threatening anemia with ongoing hemolysis and use extended matched red cellsSupportive CareErythropoietin with or without IV iron

## 6. Conclusions

In conclusion, managing HS in SCD remains a critical and evolving challenge within hematology. This literature review has underscored the complexity of diagnosing and treating HS, highlighting the necessity for early identification and a nuanced therapeutic approach. Despite the high prevalence of SCD and the significant clinical burden posed by HS, current treatment paradigms are largely empirical, stressing the need for robust clinical trials to establish evidence-based protocols.

Key strategies in managing HS include using immunosuppressive agents such as steroids, intravenous immunoglobulins (IVIG), and rituximab, which have shown varying degrees of efficacy in reducing hemolysis. However, these treatments carry risks, particularly for SCD patients, who may experience complications like rebound pain and an increased susceptibility to infections. Novel therapeutic approaches, including eculizumab, tocilizumab, plasma exchange, and erythropoietin-stimulating agents, offer promising avenues but require further validation through comprehensive research to understand their safety and effectiveness thoroughly.

The intricacies of HS pathophysiology—ranging from macrophage-mediated hemolysis to antibody-mediated delayed reactions—necessitate a tailored and dynamic management plan for each patient. Early diagnosis, careful monitoring, and a personalized treatment approach are crucial for optimizing patient outcomes. The development of biomarkers, such as soluble C5b-9, and the exploration of alternative therapies, such as Hemopure and bortezomib, represent significant steps forward in addressing the unique challenges of HS in SCD.

An enhanced understanding of the underlying mechanisms and the establishment of standardized treatment protocols through rigorous clinical trials will be essential to improving the prognosis and quality of life for patients suffering from this severe complication of SCD.

## Figures and Tables

**Table 1:** Summary of Pathogenesis of HS in Sickle Cell Patients.

Mechanism	Description
Alloantibodies	Antibodies develop against antigens on donor red blood cells (RBCs) from multiple prior transfusions
Bystander Hemolysis	Antibodies target foreign proteins on transfused cells and also damage nearby healthy RBCs in a collateral effect due to the fragility of sickle-shaped RBCs.
Macrophage Activation	Increased adhesion of sickle RBCs to macrophages leads to phagocytosis and destruction.
Complement-mediated Damage	Defective regulation of the membrane attack complex (MAC) on RBCs of SCA patients leads to complement-mediated destruction of both transfused and autologous RBCs.
HLA-mediated damage	Transfused red cells expressing HLA are targeted and destroyed by HLA-mediated immune mechanisms involving hyperactive macrophages, while those transfused cells that do not express HLA are destroyed via a bystander mechanism
Alloantibody-negative HS	Direct RBC–macrophage interaction Involvement of cytokine and complement pathways (potentially to a greater extent)

**Table 2:** Mechanisms of macrophage activation contributing to HS in sickle cell patients.

Macrophage Hyperactivation Mechanisms
* Altered CD47 signaling (reduced “don’t eat me” signal) * Increased adhesion of HbS RBCs * Increased adhesion molecule expression on RBCs (phosphatidylserine, α4β1) * Macrophage activation by hemoglobin–haptoglobin complexes

**Table 3:** Real world observational studies focusing on the treatment of HS.

Author	Age/Sex/Sickle Cell Variant	Scenario	Time to Develop Hemolysis	Steroids	IVIG	Tocilizumab	Other	Time to Normalize Hb
Silvapalaratnam et al. [3]	33/M/HbSS	Post-Exchange Transfusion (ET)	5 days	Yes	Yes	Yes		13 days
Epstein et al. [8]	19/M/HbSS	Post-Partial ET	2 days	Yes	No	No	Darbepoetin Rituximab Bortezomib Hempure	15 days
Win et al. [7]	26/M	Blood Transfusion (BT)	<24 h	No	No	No	Deceased	
McGleenan et al. [14]	32/F	BT and Surgery	4 weeks	Yes	Yes	No		30 days
Win et al. [10]	36/M	BT	3 days	Yes	Yes	No		
	36/F	BT	4 days	Yes	Yes	No		16 days
Cullis et al. [21]	32/F/HbSS	ET	2 days	Yes	Yes	No		9 days
Desai et al. [15]	21/M/HbSS	BT	5 days	Yes	Yes	Yes		26 days
	30/F/HbSS	ET	6 days	Yes	Yes		Eculizumab Pre Rituxan/Steroids	18 days
Daly et al. [22]	30/F	BT	4 days	Yes	Yes		Eculizumab	
Anderson et al. [23]	37/M	ET	8 days	Yes	Yes		Erythropoietin	
D’Amico et al. [24]	53/F	ET	3 days		Yes		Erythropoietin	18 days
Ragheb et al. [16]	38/F	BT	7 days	Yes	Yes		Eculizumab	
	40/M	BT	2 days	Yes	Yes		Eculizumab	
	45/M	BT	3 days	Yes	Yes		Eculizumab Rituximab	
Kirui et al. [25]	29/F/Hbss	BT	7 days	Yes	Yes		Eculizumab	
Asnawi et al. [26]	32/F/HbSS	BT	8 days				Deceased	

## Data Availability

Not applicable.
